# Comparison of the Efficacy of Everolimus-Eluting Stents and Paclitaxel-Eluting Balloon Angioplasty for Coronary In-Stent Restenosis: A Systematic Review and Meta-Analysis

**DOI:** 10.31083/RCM26387

**Published:** 2025-06-16

**Authors:** Zhili Wei, Ziran Luo, Yixvan Chang, Zhijing An, Sai Jin, Jianke Rong, Bing Song

**Affiliations:** ^1^The First Clinical Medical College of Lanzhou University, 730000 Lanzhou, Gansu, China; ^2^Department of Cardiovascular Surgery, First Hospital of Lanzhou University, 730000 Lanzhou, Gansu, China

**Keywords:** in-stent restenosis, paclitaxel-coated balloon, everolimus-eluting stent, systematic review/meta-analysis

## Abstract

**Background:**

The aim of the study was to systematically evaluate and compare the efficacy of everolimus-eluting stents (EESs) and paclitaxel-coated balloons (PCBs) in treating patients with in-stent restenosis (ISR).

**Methods:**

We performed a comprehensive search of the PubMed, Cochrane Library, Web of Science, and Embase databases up to August 2024. Two researchers independently conducted literature retrieval, screening, data inclusion, and quality assessment. A collaborative meta-analysis was performed using Stata 17.0.

**Results:**

A total of ten randomized controlled trials (RCTs) were included, all assessed using the Cochrane quality assessment tool and were categorized as having a low risk of bias. The analysis revealed a significantly higher need for target lesion revascularization in the PCB group compared to the EES group (odds ratio (OR) *= *2.74, 95% confidence interval (CI) (1.80–4.16),* p < *0.001, *I^2^* = 38.6%). There were no significant differences between the EES or PCB treated ISR patients in terms of all-cause mortality, cardiac death, myocardial infarction, target lesion revascularization, and stent thrombosis within one year. Subgroup analyses based on ISR causative factors showed consistent results with overall findings and significantly reduced heterogeneity.

**Conclusion:**

PCBs are associated with a higher frequency of target lesion revascularization compared to EES in the treatment of ISR. However, there are no significant differences in other outcome indicators. Therefore, EES is recommended as the preferred treatment for ISR in clinical decision-making.

**The INPLASY registration:**

INPLASY202480079, https://inplasy.com/inplasy-2024-8-0079/.

## 1. Background

Percutaneous coronary intervention (PCI) is primarily used to treat severe coronary artery stenosis [1, 2, 3], often involving the implantation of bare-metal stents (BMSs) or drug-eluting stents (DESs). While BMSs are widely used, they frequently lead to intimal hyperplasia, which can result in in-stent restenosis (ISR). Although DESs are designed to inhibit neointimal proliferation, restenosis remains a persistent challenge [4, 5]. Therefore, the treatment of ISR patients is a significant clinical burden [6, 7]. In clinical practice, ISR is characterized by the development of neovascular lesions at the stent edges or within the stent, typically occupying more than 50% of the vessel’s diameter. This condition remains a significant limitation of PCI [6, 8]. Despite its relatively low incidence, ISR remains a significant issue, especially as PCI is applied to more high-risk patients and complex lesions. Reports indicate that 5% to 20% of patients experience restenosis [7, 9, 10, 11, 12], which, although often considered benign, is associated with a higher incidence of myocardial infarction [13]. Consequently, it is crucial to focus on the treatment of ISR patients. Current approaches for treating ISR include traditional balloon angioplasty, laser atherectomy, re-implantation of either a BMS or DES, and drug-coated balloon (DCB) angioplasty [6].

Despite the array of available technologies for treating ISR patients, determining the optimal treatment method for ISR remains unsolved [6, 7, 14, 15]. Recent clinical trial guidelines suggest that both DESs and DCBs are recommended for treating ISR [6, 16, 17, 18, 19, 20, 21]. The DES are coated with antiproliferative drugs such as everolimus, zotarolimus, or paclitaxel [22]. Upon implantation, the attached drugs are gradually released, effectively inhibiting neointimal proliferation [23]. Currently, second-generation DESs offer improved efficacy compared to earlier models. Similarly, DCB surfaces are also coated with antiproliferative drugs, such as paclitaxel, but they deliver the drug directly to the lesion site without the need for a permanent stent. This targeted drug delivery helps control intimal proliferation while minimizing the risks associated with stent implantation. Studies have shown that DES treatment yields better angiographic and clinical outcomes compared to DCB treatment, primarily due to a reduced need for repeat vascular reconstruction [17, 18]. The primary limitations of DESs include the risk of stent thrombosis and secondary stenosis, attributed to their multiple metal layers. In contrast, DCB treatment addresses these concerns by reducing late lumen loss [24, 25]. Despite these advantages, the optimal strategy for treating ISR remains unresolved. This study aimed to compare the short-term (1-year) and long-term (3-year) efficacy of paclitaxel-coated balloon (PCB) catheters (Sequent Please PEB, B. Braun, Melsungen, Germany) and everolimus-eluting stents (EESs) (XIENCE, Abbott Vascular, Santa Clara, CA) in the treatment of ISR through a systematic review and meta-analysis, and to provide evidence-based guidance for clinical treatment strategies.

## 2. Methods

This study was conducted according to the internationally recognized guidelines outlined in the “Preferred Reporting Items for Systematic Reviews and Meta-Analyses (PRISMA)” [26]. It has been registered on the INPLASY platform, an international registry for research protocols. The registration includes a complete research protocol, with the registration number INPLASY202480079. This study is available at: https://inplasy.com/inplasy-2024-8-0079/, last accessed on August 16, 2024.

### 2.1 Search Strategy 

We conducted a comprehensive literature search on the treatment of ISR with PCBs or EESs across multiple databases, including PubMed, EMbase, Cochrane Library, and Web of Science. The search included studies published up to August 2024. The search utilized a combination of MeSH terms and free-text keywords. To ensure a thorough search, additional manual searches were performed. The search terms included (“in-stent restenosis” OR “obstruction of the stent”) AND (“paclitaxel-coated balloon” OR “drug-coated balloon”) AND (“everolimus-eluting stent” OR “drug-eluting stent”).

### 2.2 Study Selection

Two researchers independently screened the literature and extracted data, with cross-verification of results to ensure accuracy. Disagreements between the researchers were resolved either through discussion or by consulting a third party. The inclusion criteria for this meta-analysis were as follows: (1) The study type was limited to randomized controlled trials (RCTs); (2) Studies included patients who developed ISR following PCI; (3) Interventions included the use of PCB or EES for the treatment of ISR; (4) The study focused on outcomes including one-year all-cause mortality, cardiac death, myocardial infarction, target lesion revascularization, target vessel revascularization, and stent thrombosis. Exclusion criteria included: (1) Studies that were duplicate publications; (2) Studies that did not report relevant outcome measures; (3) Studies for which the full text could not be accessed; (4) Other meta-analyses, reviews, or letters; (5) Studies that were non-clinical, such as animal or cell experiments.

### 2.3 Study Endpoints

The primary endpoint of our study was all-cause mortality at one year post-surgery. Secondary endpoints, also assessed at one-year post-surgery, included cardiac mortality, myocardial infarction, target lesion revascularization, target vessel revascularization, and stent thrombosis.

### 2.4 Data Extraction 

Standardized data tables were utilized to extract baseline patient information and research data, including: (1) General information such as study type, first author, the study’s year and region, number of patients, age range, and sex distribution. (2) Past complications, including diabetes, insulin-dependent diabetes, hypertension, hyperlipidemia, smoking history, previous myocardial infarction, stable angina, unstable angina, and chronic renal failure. (3) Preoperative data such as left ventricular ejection fraction, which indicates heart function.

### 2.5 Quality Assessment 

All included studies were RCTs. The quality assessment was conducted using the Cochrane quality assessment tool [27]. The assessment criteria included selection, implementation, measurement, follow-up, and other biases. Specifically, it assessed whether sequence generation was random, allocation concealment was preserved, blinding of participants and outcome assessors was implemented, data were complete and accounted for, there was selective reporting, and other potential biases. Each bias is categorized as “low risk”, “unclear risk”, or “high risk”. Additionally, we utilized the Grading of Recommendations Assessment, Development and Evaluation (GRADE) methodology for evidence grading to assess the quality of evidence, including risk of bias, inconsistency, indirectness, imprecision, and publication bias, resulting in final grades of “high”, “moderate”, or “low” quality.

### 2.6 Statistical Analysis

Data analysis was conducted using Stata 17.0 software (Stata Corp LLC, College Station, TX, USA). For categorical variables, the odds ratio (OR) was utilized as the effect size, accompanied by a 95% confidence interval (CI). For continuous variables, the standard mean difference (SMD) was used as the effect size. Statistical significance was defined as a *p*-value of less than 0.05. The χ^2^ test, in combination with *I*^2^ quantification, was used to assess heterogeneity. The significance level for the *χ^2^* test was set at *α*= 0.1. If significant heterogeneity was observed, indicated by an *I*^2^ of ≥50% or a *p*-value < 0.05, a random-effects model was applied for the meta-analysis. In cases where *I*^2^ was <50% and the *p*-value was >0.05, a fixed-effects model was used. Sensitivity analysis was performed to assess the stability of the results. Subgroup analyses were conducted to further explore sources of heterogeneity. Publication bias was assessed using funnel plots and Egger’s test.

## 3. Results 

### 3.1 Study Selection 

**Fig. 1.**
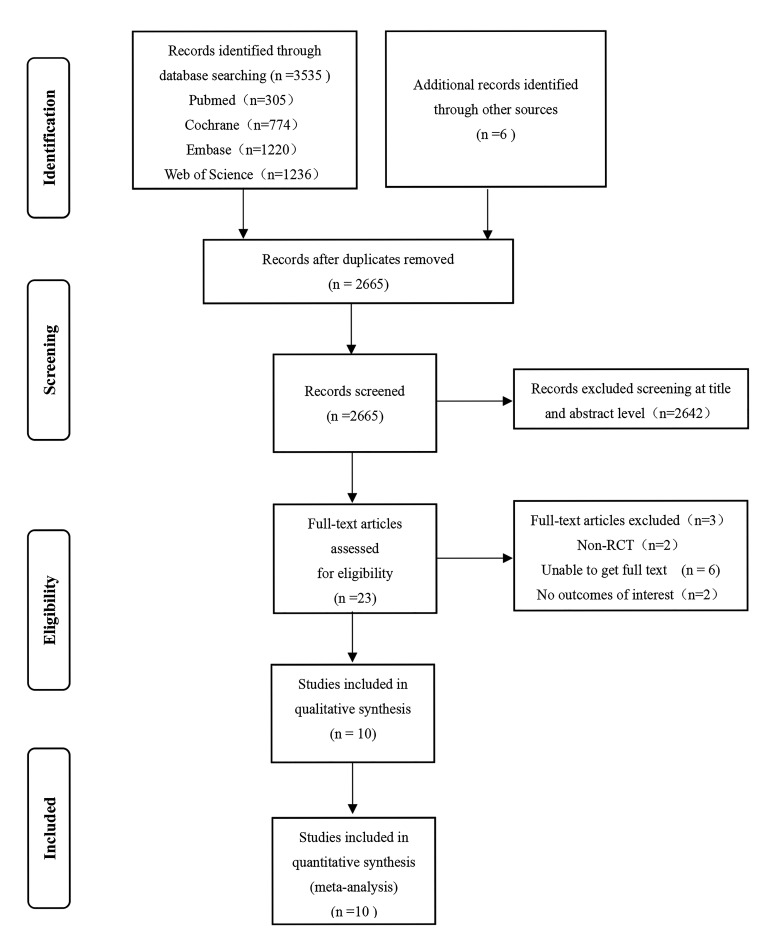
**Flow chart of the publication selection process**. RCT, randomized controlled trial.

A total of 3535 articles were initially retrieved, with an additional 6 obtained from other sources. After deduplication using EndNote 20.0 (Thomson ResearchSoft, Stanford, Connecticut, USA) and further manual processing, 2665 articles were included. Following the screening of titles and abstracts, 23 articles met the criteria. Following the full-text review, 13 articles were excluded for the following reasons: 2 were not RCTs, 6 were inaccessible in full-text, 2 did not report the outcomes of interest, and 3 exclude full text articles. Ultimately, 10 articles [13, 23, 28, 29, 30, 31, 32, 33, 34, 35], including a total of 1981 patients, were included in the analysis. The article screening process is illustrated in Fig. 1.

### 3.2 Research Characteristics and Quality Evaluation 

**Fig. 2.**
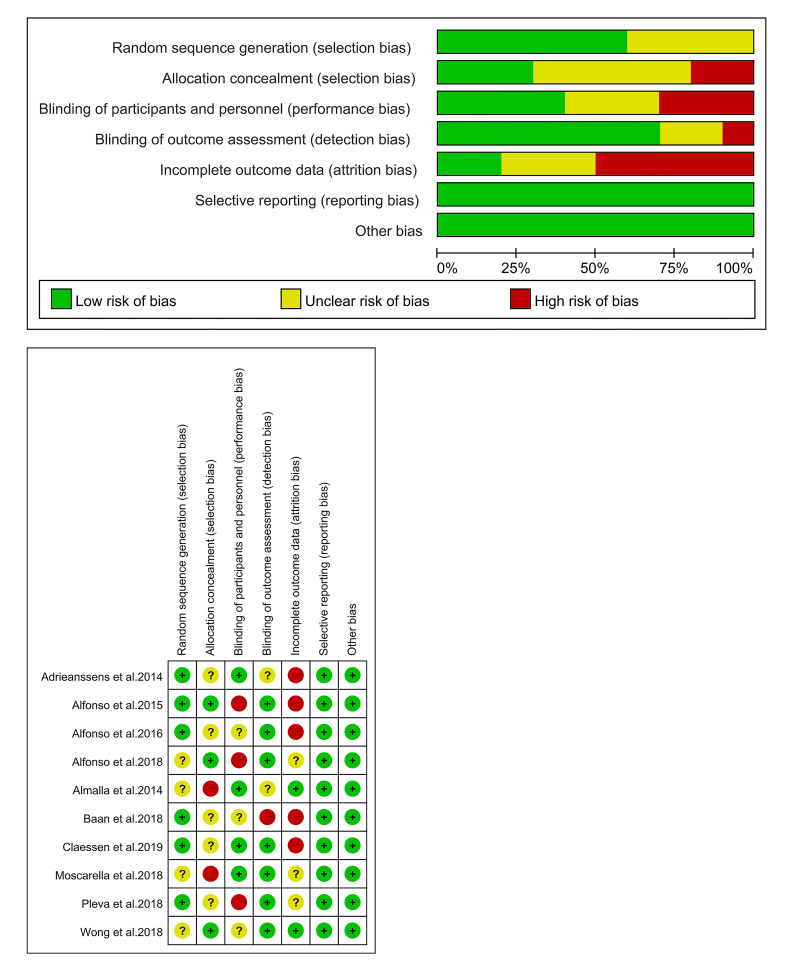
**Included study bias risk assessment results**.

All of the selected studies were RCTs published exclusively between 2014 and 2018. All studies were assessed using the Cochrane quality assessment tool, which determined that most outcome indicators are of medium to high quality, demonstrating a high level of credibility. Details of this assessment are shown in Fig. 2. Additionally, the GRADE system, which assesses the quality of evidence and strength of recommendations, classified most studies as medium to high quality. The classification details are provided in Table 1.

**Table 1.** **GRADE quality assessment**.

Certainty assessment							№ of patients	Certainty
№ of studies	Study design	Risk of bias	Inconsistency	Indirectness	Imprecision	Publication bias		
One-year all-cause mortality								
8	RCT	Not serious	Not serious	Not serious	Not serious	Undetected	1845	High
Cardiac death								
7	RCT	Serious	Not serious	Not serious	Not serious	Undetected	1674	Moderate
Myocardial infarct								
10	RCT	Not serious	Not serious	Not serious	Not serious	Undetected	1981	High
Target lesion revascularization								
7	RCT	Not serious	Not serious	Not serious	Serious	Undetected	1479	Moderate
Target vessel revascularization								
9	RCT	Not serious	Not serious	Not serious	Not serious	Undetected	1805	High
Stent thrombosis								
5	RCT	Very serious	Not serious	Not serious	Not serious	Undetected	1395	Low

RCT, randomized controlled trial; GRADE, the Grading of Recommendations Assessment, Development and Evaluation.

### 3.3 Patient Characteristics 

The baseline characteristics of the patients included in this study are shown in Table 2 (Ref. [13, 23, 28, 29, 30, 31, 32, 33, 34, 35]). A total of 1981 ISR patients were treated with PCBs or EESs. The average age of the patients was 66.1 years, with a range of 64.0 to 69.6 years. The cohort comprised 63.0% males, although the percentage varied significantly across subgroups, ranging from 15.0% to 100%. Among the patients, 35.6% had diabetes, with the prevalence ranging from 4.0% to 50.0%. Of these, 14.3% had type 1 diabetes, with a range of 10.0% to 54.0%. Hypertension was present in 61.3% of the patients, with a prevalence range from 60.0% to 85.0%. Hyperlipidemia was reported in 69.4%, with a range of 52.0% to 96.0%. Additionally, 46.2% had a history of smoking (with a range of 12.0% to 75.0%), 51.6% had a history of myocardial infarction (ranging from 25.6% to 72.8%), 49.1% had stable angina (with a range of 26.5% to 68.0%), 44.2% had unstable angina (ranging from 20.0% to 52.0%), and 10.1% had chronic renal failure (with a range of 2.9% to 26.0%).

**Table 2.** **The baseline characteristics of the patients (PCB/EES)**.

Study	General characteristics					Previous complications (%)									Preoperative examination results
	Country	Type	Total	Male (%)	Age (Y)	DM	IDDM	HTN	HL	Smoked	Previous MI	SA	UA	CRF	LVEF (%)
Adriaenssens *et al*. 2014 [35]	Belgium	RCT	49	72.0/100.0	67.6/64.2	24.0/4.0	NR	64.0/60.0	96.0/96.0	20.8/12.0	48.0/40.0	52.0/68.0	20.0/20.0	NR	NR
Alfonso *et al*. 2016 [34]	Spain	RCT	498	16.0/15.0	67.0/65.0	42.0/34.0	14.0/14.0	72.0/76.0	72.0/74.0	58.0/63.0	52.0/53.0	53.0/51.0	47.0/49.0	NR	58.0/59.0
Alfonso *et al*. 2016 [33]	Spain	RCT	189	86.0/87.0	67.0/64.0	32.0/20.0	NR	72.0/72.0	73.0/66.0	59.0/75.0	NR	60.0/55.0	40.0/45.0	NR	58.0/59.0
Alfonso *et al*. 2018 [23]	Spain	RCT	309	82.0/84.0	66.0/66.0	49.0/43.0	NR	71.0/78.0	71.0/78.0	58.0/56.0	NR	48.0/49.0	52.0/51.0	NR	NR
Almalla *et al*. 2014 [32]	Germany	RCT	86	82.0/70.0	69.6/67.7	39.1/35.0	21.7/17.5	80.4/85.0	NR	30.4/52.5	36.9/52.5	NR	NR	26.0/12.5	NR
Baan *et al*. 2018 [31]	Netherlands	RCT	278	72.0/84.0	66.0/65.0	31.0/33.0	10.0/18.0	64.0/67.0	59.0/60.0	17.0/13.0	53.0/52.0	NR	44.0/42.0	6.6/7.1	NR
Claessen *et al*. 2019 [13]	Netherlands	RCT	88	76.0/78.0	68.0/67.0	NR	33.0/54.0	69.0/67.0	74.0/52.0	17.0/15.0	57.0/61.0	67.0/52.0	NR	12.0/20.0	NR
Moscarella *et al*. 2018 [30]	Italy	RCT	176	82.0/83.0	65.8/66.5	26.2/31.5	10.7/17.4	76.2/76.1	72.1/71.4	42.9/44.6	54.8/72.8	NR	35.7/39.1	14.3/6.5	52.4/50.9
Pleva *et al*. 2018 [28]	Czech Republic	RCT	136	63.2/67.6	65.6/65.5	25.0/26.5	NR	NR	NR	45.6/42.6	63.2/60.3	33.8/26.5	NR	2.9/10.3	NR
Wong *et al*. 2018 [29]	America	RCT	172	70.9/72.1	67.0/66.0	50.0/44.2	NR	69.8/75.6	57.0/61.6	46.5/43.0	30.2/25.6	39.5/41.9	45.3/38.4	NR	59.4/59.9

Y, year; PCB, paclitaxel-eluting balloon; EES, everolimus-eluting stents; DM, diabetes mellitus; IDDM, insulin-dependent diabetes mellitus; HTN, hypertension; HL, hyperlipidemia; MI, myocardial infarction; SA, stable angina; UA, unstable angina; CRF, chronic renal failure; LVEF, left ventricular ejection fraction; NR, not report.

### 3.4 Meta-Analysis Results

#### 3.4.1 One-Year All-Cause Mortality

**Fig. 3.**
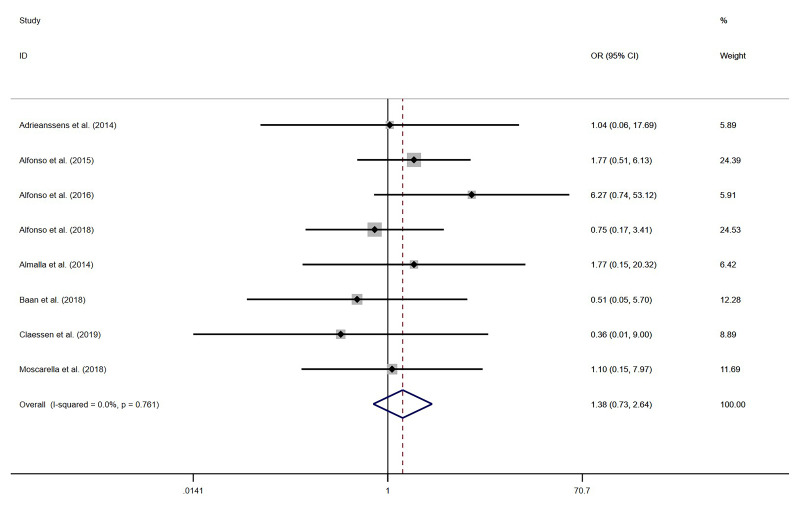
**Forest plot of one-year all-cause mortality**. OR, odds ratio; CI, confidence interval.

Eight studies [13, 23, 30, 31, 32, 33, 34, 35] reported data on one-year all-cause mortality. Given the minimal heterogeneity observed among the studies (*I*^2^ = 0.0%), a fixed-effect model was utilized for the meta-analysis. The results indicated no statistically significant difference in one-year mortality rates between the PCB group and the EES group (OR = 1.38, 95% CI (0.73–2.64), *p* = 0.322, *I*^2^ = 0.0%). Details are illustrated in Fig. 3.

#### 3.4.2 Cardiac Death 

Seven studies [13, 23, 28, 30, 31, 33, 34] reported data on cardiac death. Given the negligible heterogeneity among these studies (*I*^2^ = 0.0%), a fixed-effect model was utilized. The meta-analysis showed no statistically significant difference in cardiac death rates between the PCB group and the EES group (OR = 1.02, 95% CI (0.42–2.47), *p* = 0.963, *I*^2^ = 0.0%). Further details are provided in Table 3 (Ref. [13, 23, 28, 29, 30, 31, 32, 33, 34, 35]).

**Table 3.** **Secondary outcome measures**.

Outcomes		Studies	Heterogeneity test results		Effect model	Meta analysis results	
			*p-*value	*I*^2^ (%)		OR (95% CI)	*p*-value
Cardiac death		7 [13, 23, 28, 30, 31, 33, 34]	0.970	0.0	Fixed effect	1.02 (0.42, 2.47)	0.963
Myocardial infarct		10 [13, 23, 28, 29, 30, 31, 32, 33, 34, 35]	0.892	0.0	Fixed effect	0.97 (0.57, 1.66)	0.915
	BMS-ISR	3 [28, 33, 35]	0.868	0.0	Fixed effect	0.72 (0.24, 2.22)	0.571
	DES-ISR	3 [23, 29, 32]	0.265	0.0	Fixed effect	0.97 (0.34, 2.80)	0.960
Target lesion revascularization		7 [23, 29, 30, 32, 33, 34, 35]	0.135	38.6	Fixed effect	2.74 (1.80, 4.16)	0.000
	BMS-ISR	2 [33, 35]	0.335	0.0	Fixed effect	3.57 (1.12, 11.38)	0.031
	DES-ISR	3 [23, 29, 32]	0.243	19.8	Fixed effect	2.85 (1.47, 5.52)	0.002
Target vessel revascularisation		9 [13, 23, 28, 29, 31, 32, 33, 34, 35]	0.017	56.9	Random effect	1.14 (0.65, 2.02)	0.643
	BMS-ISR	3 [28, 33, 35]	0.129	51.2	Random effect	0.78 (0.24, 2.48)	0.671
	DES-ISR	3 [23, 29, 32]	0.012	11.2	Random effect	1.27 (0.25, 6.54)	0.772
Stent thrombosis		5 [23, 28, 32, 33, 35]	0.723	0.0	Fixed effect	1.15 (0.38, 3.45)	0.800
	BMS-ISR	3 [28, 33, 35]	0.552	0.0	Fixed effect	1.42 (0.28, 7.36)	0.673
	DES-ISR	2 [23, 30]	0.372	0.0	Fixed effect	0.97 (0.22, 4.27)	0.964

BMS-ISR, bare-metal stents-in-stent restenosis; DES-ISR, drug-eluting stents-in-stent restenosis.

#### 3.4.3 Myocardial Infarction

A total of 10 studies [13, 23, 28, 29, 30, 31, 32, 33, 34, 35] reported on myocardial infarction. Due to the absence of significant heterogeneity among the studies (*I*^2^ = 0.0%), a fixed-effect model was used for the meta-analysis. The meta-analysis revealed no statistically significant difference between the PCB-treated group and the EES-treated group (OR* = *0.97, 95% CI (0.57–1.66), *p = *0.915, *I*^2^ = 0.0%). For further details, refer to Table 3.

#### 3.4.4 Target Lesion Revascularization 

We analyzed data from seven studies [23, 29, 30, 32, 33, 34, 35] on target lesion revascularization using a fixed-effect model, as heterogeneity among the studies was not significant (*I*^2^ = 38.6%). The meta-analysis indicated a significantly higher probability of target lesion revascularization in the PCB group compared to the EES group (OR* = *2.74, 95% CI (1.80–4.16), *p* < 0.001, *I*^2^ = 38.6%). Detailed results can be found in Table 3.

#### 3.4.5 Target Vessel Revascularization

Nine studies [13, 23, 28, 29, 31, 32, 33, 34, 35] provided data on target vessel revascularization. Due to significant heterogeneity among the studies (*I*^2^ = 56.9%), a random-effects model was used for analysis. The results indicated no statistically significant difference between the PCB and EES groups (OR* = *1.14, 95% CI (0.65–2.02), *p* = 0.643, *I*^2^ = 56.9%). Detailed findings are shown in Table 3.

#### 3.4.6 Stent Thrombosis 

Results related to stent thrombosis were reported by five studies [23, 28, 32, 33, 35]. Given the negligible heterogeneity among the studies (*I*^2^ = 0.0%), a fixed-effect model was applied. The meta-analysis showed no statistically significant difference between the PCB group and the EES group (OR* = *1.15, 95% CI (0.38–3.45), *p* = 0.800, *I*^2^ = 0.0%). For further details, refer to Table 3.

### 3.5 Subgroup Analysis 

Based on the cases of ISR, further analyses were conducted to explore sources of significant heterogeneity observed in certain outcome measures. These analyses were divided into two subgroups: Bare-metal stent-in-stent restenosis (BMS-ISR) and drug-eluting stent-in-stent restenosis (DES-ISR). The results of the one-year all-cause mortality subgroup analysis are as described below. The subgroup analyses of other secondary outcome measures were consistent with the overall results. For detailed results, refer to Table 3.

#### One-Year Overall Mortality Rate Subgroup Analysis Regarding ISR Causes

In the one-year mortality rate analysis, the BMS-ISR subgroup, which included three studies [31, 33, 35], was analyzed separately using a fixed effects model. This analysis revealed no statistically significant difference between the PCB group and the EES group (OR = 2.05, 95% CI (0.61–6.92), *p = *0.245, *I*^2^ = 0.0%). Similarly, the DES-ISR subgroup, which also comprised three studies [23, 30, 32], was analyzed separately using a fixed effects model. This analysis demonstrated no statistically significant difference between the PCB group and the EES group (OR = 1.00, 95% CI (0.35–2.89), *p* = 0.999, *I*^2^ = 0.0%).

### 3.6 Sensitivity Analysis

**Fig. 4.**
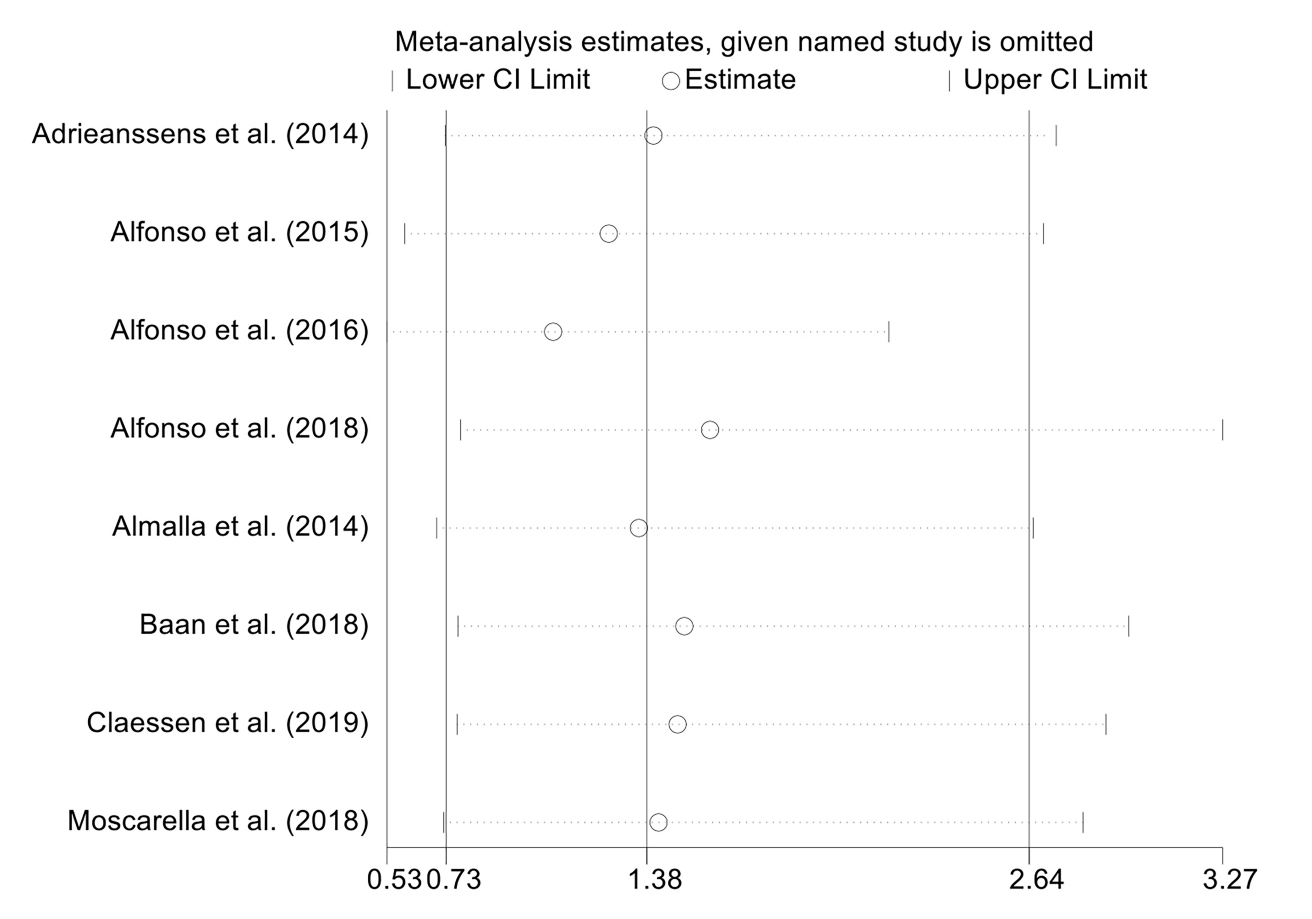
**Sensitivity analysis of the one-year all-cause mortality rate**.

Sensitivity analysis was performed by sequentially excluding individual studies. This approach confirmed that the results remained stable and showed no significant changes. The one-year all-cause mortality rate is illustrated in Fig. 4.

### 3.7 Bias in Published Results

**Fig. 5.**
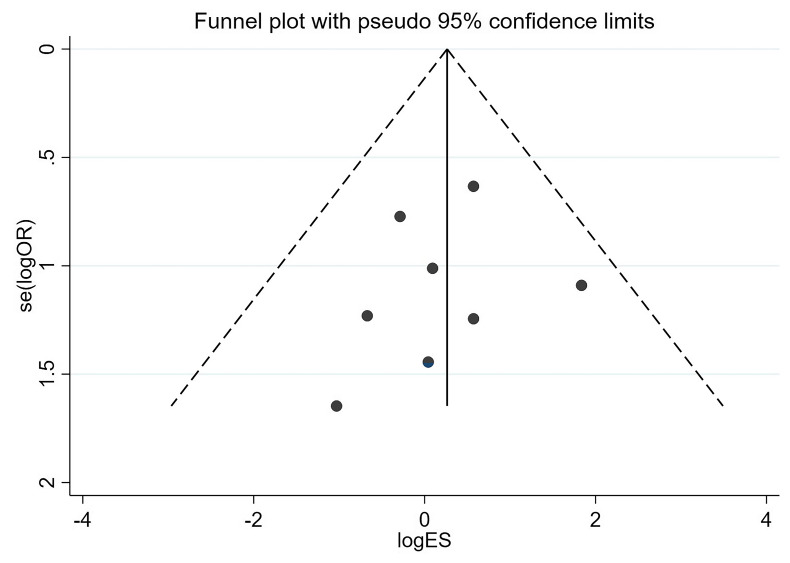
**Funnel plots of the one-year all-cause mortality rate**. ES, effect size.

**Fig. 6.**
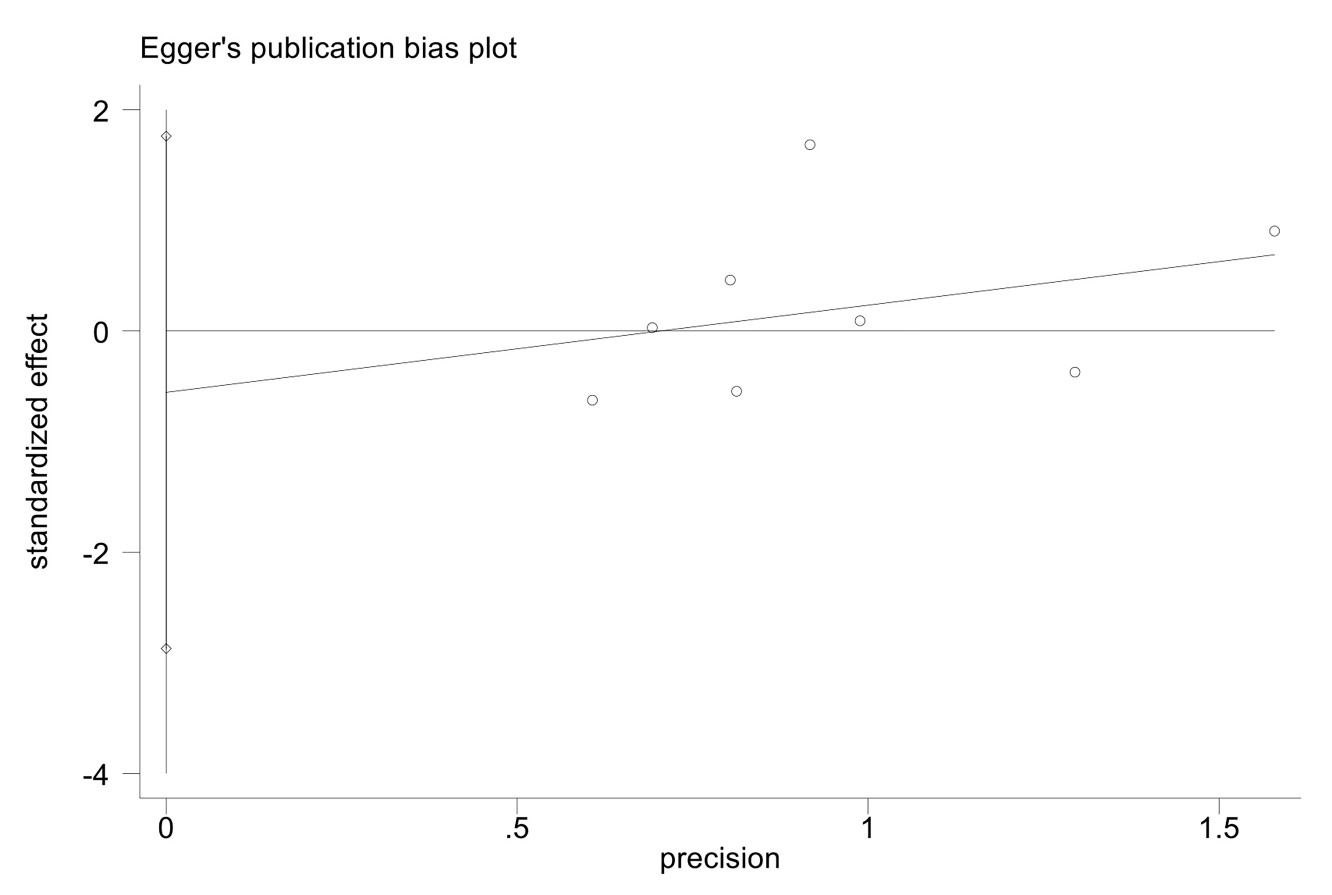
**Egger’s regression test of the one-year all-cause mortality rate**.

We conducted a qualitative risk of bias assessment using funnel plots for all outcome indicators, and the results indicate that the funnel plots were symmetrical for each outcome. Fig. 5 illustrates the one-year all-cause mortality rate. Subsequently, we conducted a quantitative assessment of bias risk using Egger’s regression test. The one-year all-cause mortality rate is illustrated in Fig. 6. The results were as follows: One-year all-cause mortality rate (*p = *0.579), Cardiogenic death (*p* = 0.081), Myocardial infarction (*p* = 0.403), Target lesion revascularization (*p* = 0.813), Target vessel revascularization (*p* = 0.236), Stent thrombosis (*p = *0.668). These results suggest that there was no statistically significant publication bias across any of the measured outcomes.

## 4. Discussion

As PCI becomes more widely used, ISR has emerged as a major concern and limitation of the procedure [6, 8], drawing increasing attention in clinical practice. Moreover, as lesions become more complex, the incidence of restenosis is also rising. Research suggests that ISR is often a consequence of inevitable damage to the vascular intima during stent implantation, which triggers the proliferation of neointima. During this process, smooth muscle cells, facilitated by adhesion molecules, migrate to the injury site to form neointima [36, 37, 38] and secrete cytokines that further promote intimal proliferation [39]. These cytokines enhance the role of the smooth muscle cells in neointimal growth. Additionally, ISR may also be related to the formation of new atherosclerotic lesions. Studies have shown that the probability of detecting atherosclerosis increases during the later stages of ISR following stent implantation [40, 41, 42, 43]. This process is mainly characterized by the accumulation of foam cells (lipid-laden macrophages) around the stent, which subsequently leads to the formation of atheromatous plaques. The rupture of these plaques is a direct cause of ISR. Additionally, vascular damage triggers coagulation and enhances platelet activity, which not only promotes neointimal growth but also significantly raises the risk of thrombosis, potentially contributing to ISR. Currently, the optimal treatment method for ISR remains undetermined, with DES and DCB being the primary approach. EESs, a type of DES, are among the most commonly used and effective options, while paclitaxel is the most commonly used drug in DCB treatments. Although EESs are effective for treating various types of ISR [44], studies have shown that patients may still experience restenosis after treatment, and resistance to everolimus can impact its therapeutic effect. Furthermore, studies indicate that PCB treatments lead to significantly lower rates of late lumen loss compared to EES treatments. This is due to the absence of the need for repeated stent implantation [13, 45], which also improves their effectiveness in inhibiting neointimal proliferation. Given the optimal treatment method for ISR has not yet been determined [6, 7, 14, 15], it is essential to compare the efficacy of EES and DCB in treating ISR patients. Such comparisons will provide valuable insights for clinical practice.

A 2018 study by Alfonso *et al*. [23] indicated that the likelihood of requiring target lesion revascularization in ISR patients treated with PCB was significantly higher than in those treated with EES (OR = 3.16, *p = *0.007). A 2014 study by Adriaenssens* et al*. [35] found no significant difference in the probability of myocardial infarction between ISR patients treated with either PCB or EES (OR = 0.33, *p* = 0.320). In 2018, a study by Baan *et al*. [31] also showed no significant difference in the probability of cardiogenic death between the ISR patients treated with either PCB or EES (OR = 0.34, *p* = 0.320). In 2020, Giacoppo D *et al*. [44] conducted a systematic review comparing the efficacy of DCB and DES in treating ISR patients. However, the DCB and DES included a wide variety of types, and no separate comparison was made between PCB and EES. In 2024, Guo S *et al*. [46] carried out a network meta-analysis comparing various treatment measures for ISR. However, the limitation was that when comparing PCB and EES, the selection of endpoint indicators was limited, and could not fully reflect the differences between the two treatment measures. These findings indicate inconsistent conclusions regarding the efficacy of PCB versus EES in treating ISR. Therefore, this study synthesizes past research to compare the efficacy differences of PCB and EES in treating ISR patients. The meta-analysis results show that in terms of target lesion revascularization, the likelihood of requiring this procedure was significantly greater in the PCB group compared to the EES group, consistent with previous findings [23, 34]. Potential contributing factors are: (1) For DES-ISR patients, there is an increased probability of neoatherosclerosis formation, and other potential factors may affect the demand for target lesion revascularization [23]. (2) There is a heterogeneous increase in fibrin deposition and loose connective tissue [47]. (3) The antiproliferative agents eluted from EES delay vascular healing [48]. In terms of one-year all-cause mortality, cardiogenic death, myocardial infarction, target lesion revascularization, and in-stent thrombosis, no significant differences were observed between the PCB group and the EES group, consistent with previous findings [28, 29]. This can be attributed to: (1) Both PCB and EES effectively preserved some endothelial functions within the vessels. (2) Both PCB and EES exhibited excellent anti-proliferative effects, preventing excessive neointimal hyperplasia. (3) Both PCB and EES reduced the risk of thrombosis formation.

### Limitations of the Study 

The study has the following limitations: (1) The comparison involved the PCB group with a single-layer strut and the EES group with a double-layer strut. This difference in strut could have influenced the outcome indicators. (2) The exclusion of highly complex ISR cases, including those with total occlusions, small vessels, or extensively diffuse disease may mean the generalizability of these findings to ISR patient populations with more complex coronary anatomies remains uncertain. (3) The study did not consider the impact of angiographic monitoring on revascularization rates. (4) These findings are only applicable to ISR patients who underwent sufficient pre-dilation before the procedure. (5) The exclusion of studies of a non-RCT design may have contributed to heterogeneity.

## 5. Conclusion 

No significant differences were observed between the PCB and EES groups in terms of one-year all-cause mortality, myocardial infarction, and stent thrombosis; however, the PCB group required significantly more frequent revascularization of target lesions compared to the EES group. Therefore, it is recommended to preferentially use EES in the treatment of ISR for clinical decision-making. We sincerely hope that further studies regarding the long-term efficacy of EES versus PCB in the treatment of ISR will be carried out.

## Availability of Data and Materials

The datasets analyzed during the current study are available from the corresponding author on reasonable request.

## Supplementary Material

Supplementary material associated with this article can be found, in the online version, at https://doi.org/10.31083/RCM26387.
